# *Chlorella vulgaris* and Its Phycosphere in Wastewater: Microalgae-Bacteria Interactions During Nutrient Removal

**DOI:** 10.3389/fbioe.2020.557572

**Published:** 2020-09-22

**Authors:** Roland Wirth, Bernadett Pap, Tamás Böjti, Prateek Shetty, Gergely Lakatos, Zoltán Bagi, Kornél L. Kovács, Gergely Maróti

**Affiliations:** ^1^Institute of Plant Biology, Biological Research Centre, Szeged, Hungary; ^2^Department of Biotechnology, University of Szeged, Szeged, Hungary; ^3^Department of Oral Biology and Experimental Dental Research, Faculty of Dentistry, University of Szeged, Szeged, Hungary; ^4^Faculty of Water Sciences, National University of Public Service, Baja, Hungary

**Keywords:** wastewater, green algae, phycosphere, algal-bacterial interactions, metagenomics

## Abstract

Microalgae-based bioenergy production is a promising field with regard to the wide variety of algal species and metabolic potential. The use of liquid wastes as nutrient clearly improves the sustainability of microalgal biofuel production. Microalgae and bacteria have an ecological inter-kingdom relationship. This microenvironment called phycosphere has a major role in the ecosystem productivity and can be utilized both in bioremediation and biomass production. However, knowledge on the effects of indigenous bacteria on microalgal growth and the characteristics of bacterial communities associated with microalgae are limited. In this study municipal, industrial and agricultural liquid waste derivatives were used as cultivation media. *Chlorella vulgaris* green microalgae and its bacterial partners efficiently metabolized the carbon, nitrogen and phosphorous content available in these wastes. The read-based metagenomics approach revealed a diverse microbial composition at the start point of cultivations in the different types of liquid wastes. The relative abundance of the observed taxa significantly changed over the cultivation period. The genome-centric reconstruction of phycospheric bacteria further explained the observed correlations between the taxonomic composition and biomass yield of the various waste-based biodegradation systems. Functional profile investigation of the reconstructed microbes revealed a variety of relevant biological processes like organic acid oxidation and vitamin B synthesis. Thus, liquid wastes were shown to serve as valuable resources of nutrients as well as of growth promoting bacteria enabling increased microalgal biomass production.

## Introduction

Biofuels derived from microalgae are alternative second-generation biofuels having no significant impact on agriculture (Klassen et al., 2016; Rizwan et al., 2018; Wirth et al., 2018). Microalgae have a higher biomass productivity than that of terrestrial crops and can be cultivated on marginal land area all year round. Additionally, the use of microalgae have the potential to directly reduce greenhouse gas emissions (GHG) through the replacement of fossil fuels and by photosynthetic CO_2_ fixation in their biomass (Lam and Lee, 2012; Yen et al., 2013). Water and nutrients are identified as important limiting resources for microalgae production. The nutrients for microalgae cultivation are readily available in various types of wastewater. Using photoheterotrophic microalgae in biological wastewater treatment represents a dual exploitation of green algae, removing dissolved organic and inorganic pollutants is combined with the production of sustainable bioresource for biofuel production (Mujtaba et al., 2015; Guldhe et al., 2017; Cheah et al., 2018; Vo Hoang Nhat et al., 2018; Li et al., 2019; Shetty et al., 2019). Microalgae have an evolutionary determined ecological relationship with bacteria in natural aquatic environments representing an important interkingdom association (Fuentes et al., 2016). These interactions are strongly influenced by nutrient cycling which regulates the productivity and stability of natural aquatic food webs. The intimate relationship between microalgae and bacteria represents the phycosphere, a key microenvironment ultimately mediating the ecosystem productivity (Cho et al., 2015; Seymour et al., 2017). The exchange of micro- and macronutrients defines the relationship of the interactive partners, which are influenced by a number of key aspects. Firstly, the pH level and the available nutrients determine the surrounding chemical environment, which has a central role in chemotaxis, the motility of bacteria, which enables microbial colonization (Medipally et al., 2015). Secondly, the bacterial communities in the specific ecosystem have important roles in shaping the phycosphere. The most frequently observed bacteria in wastewaters are affiliated with the phyla of the *Bacteroidetes* and *Alpha*-, *Beta*-, and *Gammaproteobacteria* (with Plant Growth Promoting Bacteria (PGPB) among them) (Guo and Tong, 2014; Kouzuma and Watanabe, 2015; Calatrava et al., 2018). Thirdly, the available microalgae and bacteria synergistically affect each other’s physiology and metabolism. Microalgae produce O_2_ through photosynthesis for consumption by the actively respiring aerobic bacteria, while bacteria release CO_2_, which improves the photosynthetic efficiency of green microalgae (Mouget et al., 1995). Another important interkingdom interaction is observed between vitamin-synthetizing bacteria and vitamin auxotrophic microalgae. Most microalgae are auxotrophic for vitamin B derivatives, which are essential for growth and provided by bacteria in exchange for organic carbon (Croft et al., 2005, 2006). Fourthly, the competition for available nutrients, algicidal activities or related defense mechanisms of microalgae are important factors in phycosphere development. Similarly to other natural symbiotic settings, there is only a thin line separating mutualistic and antagonistic associations between microalgae and bacteria (Santos and Reis, 2014; Ramanan et al., 2016).

There are three main sources of wastewater intensively studied in alternative microalgal cultivation; municipal, industrial and agricultural wastewater (Chiu et al., 2015; Guldhe et al., 2017). Utilization of natural microalgal-bacterial communities is a highly promising recycle solution for liquid wastes. This inexpensive and environment-friendly system can contribute to the sustainable management of water resources (Liu J. et al., 2017; Qi et al., 2018). The green microalgae *Chlorella vulgaris* is the most investigated eukaryotic algae species in wastewater treatment (Chiu et al., 2015; Otondo et al., 2018; Shetty et al., 2019). *C. vulgaris* is a common eukaryotic microalgae species found in various natural and engineered freshwater and soil habitats. *C. vulgaris* has a relatively small cell size, thin cell wall, fast growth rate and short reproduction time. This alga is a robust strain that can easily accommodate to changing physico-chemical conditions. Under nutrient limitation and stress *C. vulgaris* often accumulate high amount of lipids as store materials. These features make this microalgae suitable to cultivate in wastewater, thereby using it for combined wastewater treatment and bioenergy generation (Mussgnug et al., 2010; Collet et al., 2011; Mahdy et al., 2014; Klassen et al., 2016, 2017). It was observed that high nitrogen and phosphorus removal efficiency can be reached with *Chlorella* species (Chiu et al., 2015; Guldhe et al., 2017; Chen et al., 2018).

A number of studies examined municipal wastewater treatment efficiency using *Chlorella*-bacteria mixed cultures (Mujtaba et al., 2015; Otondo et al., 2018). More efficient nutrient removal was observed from settled domestic wastewater compared to the commonly used activated sewage process, which indicated the potential of microalgae in the activated sludge process potentially as a secondary step for further nutrient reduction and concomitant biomass production (Otondo et al., 2018). Besides, CO_2_ originated from the degradation of carbonaceous matter in an activated sludge process is released freely into the atmosphere, thus promoting GHG accumulation. In contrast, microalgae can assimilate CO_2_ into cellular components such as lipid and carbohydrate, thus achieving pollutant reduction in a more environmental-friendly way (Santos and Reis, 2014; Gonçalves et al., 2017).

In the bioenergy industry biogas is used as a source for generation of heat and/or electricity (Mao et al., 2015; Ullah Khan et al., 2017). Besides biogas, digestate is another important byproduct of anaerobic degradation of organic wastes. Digestate processing is a major bottleneck in the development of the biogas industry. Digestate can be separated into solid (10–20%) and liquid (80–90%) fractions (Xia and Murphy, 2016). Solid digestate is easily stored and transported, and can be used as an agricultural biofertilizer. However, liquid phase processing is more difficult mostly due to its relatively high ammonia content (Uggetti et al., 2014). Digestate is continuously produced, while land application is dependent on the growth stage of the crop and the period of the year. Therefore, digestate needs to be stored, which can increase GHG emission and the general costs as well (Xia and Murphy, 2016; Zhu et al., 2016). Previous studies reported that *Chlorella* species can be applied to treat liquid digestate (Collet et al., 2011; Skorupskaite et al., 2015; Uggetti et al., 2016). The performance of treatment is dependent on the algae access to carbon, nitrogen and phosphorous as well as on the availability of photosynthetically active light, which indicates a mixotrophic algae growth (Skorupskaite et al., 2015; Zhu et al., 2016).

The rapid growth of the poultry industry in agriculture has raised the need for poultry waste treatment (Sakar et al., 2009). The runoff coming from the chicken farms is highly harmful for the environment through altering the nitrogen and phosphorus balance (Liu Q. et al., 2017). One possible treatment of chicken manure is the anaerobic degradation (Anjum et al., 2017). Chicken manure can be used in small quantities in biogas producing anaerobic fermenters. High dosage of chicken manure cause ammonia accumulation and process failure (Nie et al., 2015; Sun et al., 2016). Water extraction is one possible solution for this problem (Böjti et al., 2017). The supernatant liquid waste still contains high amount of nitrogen and phosphorus, thereby represents suitable medium for microalgal biomass production (Han et al., 2017).

From the biotechnological process point of view the goal is to strengthen the mutually beneficial algal-bacterial interactions to achieve higher biomass growth (beside the bioremediation of liquid wastes). The present study examined and compared different types of wastewater recycling processes using microalgae and their specific bacterial partners. This investigation mainly focused on the interacting bacterial members in specific liquid wastes. The ubiquitous relationship between eukaryotic microalgae and bacteria should be taken into account when designing innovations in microalgal biotechnology (Cooper and Smith, 2015; Gonçalves et al., 2017; Quijano et al., 2017; Lian et al., 2018).

## Materials and Methods

### Algal-Bacterial Biomass Cultivation on Different Types of Wastewaters

The *Chlorella vulgaris* MACC-360 microalgae was obtained from the Mosonmagyaróvár Algal Culture Collection (MACC) of Hungary. *C. vulgaris* was maintained and cultivated on TAP (Tris-acetate-phosphate) plates, then TAP liquid medium (500 mL) was used for the pre-growth of microalgal biomass. The TAP plates and liquid media were incubated at 50 μmol m^–2^ s^–1^ light intensity at 25°C for 4 days (OD_750_: 4.00 ± 0.20). The microalgal stock solution was equally distributed in 17–17 mL portions into 50 mL Falcon tubes with a final optical density (OD_750_) of 0.70 ± 0.10. Microalgal biomass was separated by centrifugation from the medium and used for inoculation (microalgal dry mass content: ∼100 mg/L). TAP medium was an internal control during the experiment. Different wastewater types were prepared as follows:

#### Chicken Manure Supernatant (CMS)

Chicken manure (CM) was collected from a commercial broiler poultry farm (Hungerit Corp.) located at Csengele, Hungary. The free-range poultry houses use wheat straw bedding. Water extraction comprised of soaking 2,5 g; 5 g; 10 g and 20 g CM in 100 mL distilled water (v/v %: 2,5; 5; 10 and 20) at room temperature followed by separation of the liquid (CMS: chicken manure supernatant) and solid phases by centrifugation (10,000 rpm for 8 min).

#### Anaerobic Fermentation Effluent (FE)

Inoculum sludge was obtained from an operating biogas plant (Zöldforrás Ltd) using pig manure and maize silage mixture as feedstock. The liquid and solid phases were separated by centrifugation (10,000 rpm for 8 min). Distilled water was used to dilute FE (2, 5, 10 and 20 mL effluent in 100 mL distilled water, respectively), to the final concentrations of 2; 5; 10 and 20% (v/v %), respectively.

#### Municipal Wastewater (MW)

The municipal wastewater was originated from the Municipal Wastewater Plant of Szeged, Hungary and sampled from the secondary settling tank. The liquid phase was separated from the solid phase by centrifugation (10,000 rpm 8 min). Final concentrations were set at 20 and 50 v/v % using distilled water. Non-diluted (100 v/v %) MW was also used for cultivation.

Cultivation was performed in 250 mL serum bottles (Wheaton glass serum bottle, WH223950) with liquid volume of 200 mL and stirred on a magnetic stirrer tray. Cultivation time was 4 days. Bottles were sealed with paper plugs. Different media were incubated at 50 μmol m^–2^ s^–1^ light intensity at 25°C. The OD_750_ values of the different wastewater media were summarized in Supplementary Information.

### Determination of Cultivation Parameters

#### DM/oDM Measurements

The dry matter (DM) content was quantified by drying the biomass at 105°C overnight and weighing the residue. Further heating of this residue at 550°C for 1 h provided the organic dry mass (oDM) content.

#### C/N Ratio

To determine C/N (both liquid and biomass), an Elementar Analyzer Vario MAX CN (Elementar Group, Hanau, Germany) was employed. The approach is based on the principle of catalytic tube combustion under O_2_ supply at high temperatures (combustion temperature: 900°C, post-combustion temperature: 900°C, reduction temperature: 830°C, column temperature: 250°C). The desired components were separated from each other using specific adsorption columns (containing Sicapent (Merck, Billerica, MA, United States), in C/N mode) and were determined in succession with a thermal conductivity detector. Helium served as flushing and carrier gas.

#### NH_4_^+^-N

For the determination of NH_4_^+^ ion content, the Merck Spectroquant Ammonium test (1.00683.0001) (Merck, Billerica, MA, United States) was used.

#### Total Phosphate Measurement

Total phosphate content of the different types of wastewater were measured by the standard 4500-PE ascorbic acid, molybdenum blue method (APHA-AWWA-WPCF, 1998).

#### VOAs (Volatile Organic Acids)

The VOAs measurement process was carried out using a Pronova FOS/TAC 2000 Version 812-09.2008 automatic titrator (Pronova, Berlin, Germany).

#### Acetate Concentration

The samples were centrifuged (13,000 rpm for 10 min) and the supernatant was filtered through polyethersulfone (PES) centrifugal filter (PES 516-0228, VWR) at 16,000 g for 20 min. The concentrations of volatile organic acids were measured with HPLC (Hitachi LaChrome Elite) equipped with refractive index detector L2490. The separation was performed on an ICSep ICE-COREGEL—64H column. The temperature of the column and detector was 50 and 41°C, respectively. 0.01 M H_2_SO_4_ (0.8 mL min^–1^) was used as eluent. Acetate, propionate, and butyrate were determined in a detection range of 0.01–10 g L^–1^. Propionate and butyrate were present in traces relative to acetate and therefore these are not reported in the results section.

#### BOD (Biological Oxygen Demand) Test

To measure the biochemical oxygen demand of the wastewater samples a 5-day BOD test was applied (OxiTop OC 110, Wissenschaftlich-Technische Werkstätten GmbH). In the parallel 500 mL BOD-sample bottles 43 mL of wastewater solution were placed. The results were read after 5 days in mg O_2_/L.

#### BMP (Biochemical Methane Potential) Test

Experiments were carried out in 160 mL reactor vessels (Wheaton glass serum bottle, Z114014 Aldrich) containing 60 mL liquid phase at mesophilic temperature (37 ± 0.50°C). All fermentations were done in triplicates. The inoculum sludge was filtered to remove particles larger than 1 mm and was used according to the VDI 4630 protocol (Vereins Deutscher Ingenieure 4630, 2006). Each batch fermentation experiment lasted for 30 days in triplicates.

#### Gas Chromatographic Analysis

The CH_4_ content was determined with an Agilent 6890N GC (Agilent Technologies) equipped with an HP Molesive 5 Å (30 m × 0.53 mm × 25 μm) column and a TCD detector. The temperature of the injector was 150°C and split mode 0.2:1 was applied. The column temperature was maintained at 60°C. The carrier gas was Linde HQ argon 5.0 with the flow rate set at 16.80 mL/min. The temperature of TCD detectore was set to 150°C.

In this study data originated from the most effective cultivations under illumination are summarized and highlighted (MW: 100 v/v %, FE: 10 v/v % and CMS: 5 v/v %). All data collected under the various dilution parameters are shown in Supplementary Information.

### Total DNA Isolation for Metagenomics

The composition of the microbial community was investigated two times during the experimental period from each wastewater type and control (TAP), i.e., at the starting point (inoculation) and at the end of cultivation. For total community DNA isolation 2 mL of samples were used from each cultivation media type. DNA extraction and quality estimation were performed according Wirth et al. (2019).

### Shotgun Sequencing

The Ion Torrent PGM^TM^ platform was used for shotgun sequencing, the manufacturer’s recommendations were followed (Life Technologies, United States). Sample preparation, quantification and barcoding were described previously (Wirth et al., 2019). Sequencing was performed with Ion PGM 200 Sequencing kit (4474004) on Ion Torrent PGM 316 chip. The characteristic fragment parameters are summarized in Supplementary Table 1. Raw sequences are available on NCBI Sequence Read Archive (SRA) under the submission number: PRJNA625695.

### Raw Sequence Filtering

Galaxy Europe server was employed to pre-process the raw sequences (i.e., sequence filtering, mapping, quality checking) (Afgan et al., 2016). Low-quality reads were filtered by Prinseq (Schmieder and Edwards, 2011) (min. length: 60; min. score: 15; quality score threshold to trim positions: 20; sliding window used to calculated quality score:1). Filtered sequences were checked with FastQC (Supplementary Table 1).

### Read-Based Metagenome Data Processing and Statistical Analysis

After filtering and checking the passed sequences were further analized by Kaiju applying default greedy run mode on Progenomes2 database (Menzel et al., 2016; Mende et al., 2017). MEGAN6 was used to investigate microbial communities and export data for statistical calculation (Huson et al., 2016). Statistical Analysis of Metagenomic Profiles (STAMP) was used to calculate principal component analysis (PCA) employing ANOVA statistical test (Parks and Beiko, 2010). The distribution of abundant microbial classes between cultivation media were presented with Circos (Krzywinski et al., 2009).

### Metagenome Co-assembly, Gene Calling and Binning

The filtered sequences produced by Prinseq were co-assembled with Megahit (Li et al., 2015) (min. contig length: 2000; min k-mer size: 21; max k-mer size: 141). Bowtie 2 was equipped to mapped back the original sequences to the contigs (Langmead and Salzberg, 2012). Then Anvi’o V5 was used following the “metagenomics” workflow (Eren et al., 2015). Briefly, during contig database generation GC content, k-mer frequencies were computed, open reading frames were identified by Prodigal (Hyatt et al., 2010) and Hidden Markov Modell (HMM) of single-copy genes were aligned by HMMER on each contig (Finn et al., 2011; Campbell et al., 2013; Rinke et al., 2013; Simão et al., 2015). InterProScan v5.31-70 was used on Pfam and Kaiju on NCBInr database for the functional and taxonomic annotation of contigs (Finn et al., 2014, 2017; Jones et al., 2014; Menzel et al., 2016). The taxonomic and functional data were imported into the contig database. BAM files made by Bowtie2 were used to profile contig database, in this way sample-specific information was obtained about the contigs (i.e., mean coverage of contigs) (Langmead and Salzberg, 2012). Three automated binning programs, namely CONCOCT, METABAT2 and MAXBIN2 were employed to reconstruct microbial genomes from the contigs (Alneberg et al., 2013; Kang et al., 2015; Wu et al., 2015). The Anvi’o human-guided binning option was used to refine MAGs Anvi’o interactive interface was employed to visualize and summarize the data. Binning statistics is summarized in Supplementary Table 1. Figure finalization was made by open-source vector graphics editor Gimp 2.10.8^1^. Prokka was employed to translate and map protein sequences (create protein FASTA file of the translated protein coding sequences) (Seemann, 2014). For the calculation of module completion ratio (MCR) MAPLE 2.3.2 (Metabolic And Physiological potentiaL Evaluator) was used (Arai et al., 2018). This automatic system is mapping genes on an individual genome and calculating the MCR in each functional module defined by Kyoto Encyclopedia of Genes and Genomes (KEGG) (Kanehisa and Guto, 2000) (Supplementary Table 2).

## Results

### Bioremediation Efficiency and Biochemical Methane Potential (BMP) of the Cultivated Algal-Bacterial Biomass

**FIGURE 1:**
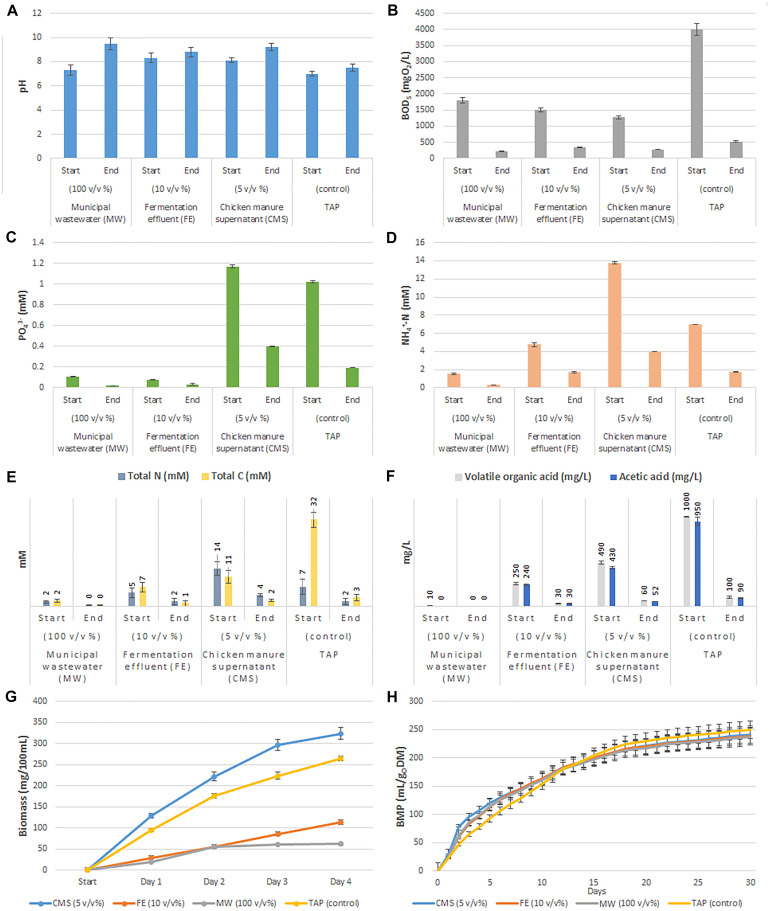
Summary of microalgal-bacterial bioremediation and cultivation efficiency on different types of wastewater. **(A)** Results of pH measurements. **(B)** Results of biological oxygen demand calculations. **(C)** Total phosphate measurements. **(D)** Ammonium ion measurement data. **(E)** Total carbon and nitrogen contents. **(F)** Volatile organic acid (VOAs) and acetic acid concentrations. **(G)** Biomass growth dynamics over time (days). **(H)** Cumulative biological methane potential of cultivated biomasses.

The bioremediation efficiency of *Chlorella vulgaris* microalgae and its phycosphere was characterized through the assessment of carbon, nitrogen, phosphate and BOD removal capability of the algal-bacterial biomass (Figure 1). The performance of microalgal-bacterial dry biomass was monitored in three liquid waste types i.e., municipal wastewater (MW), fermentation effluent (FE) and chicken manure supernatant (CMS) over 4 days. The light conditions in the cultivating media are of key importance for microalgal biomass generation. The applied wastewater types are typically dark liquids; therefore, different dilutions with distilled water were prepared in order to increase light penetration to the cultures. Only the experimental data of the most effective dilutions (non-diluted MW, 10 v/v % FE and 5 v/v % CMS) are shown and discussed in the main text of the article (efficiency was defined by the obtained yield of microalgal biomass). However, the nutrient composition of all dilutions for each liquid waste were measured and detailed in Supplementary Information. TAP medium was used as control during the experiments. Significant nutrient removal was observed in all three types of investigated wastewater indicating an active metabolism of the *C. vulgaris* microalgae and its bacterial partners. However, due to the specific features of the various liquid wastes serving as growth media the algal-bacterial nutrient removal and bioremediation capability was strongly varying. There is a clear correlation between the available nutrients (phosphate, nitrogen and acetate) and the algal-bacterial biomass yield.

The non-diluted municipal wastewater (MW) originated from the second settling tank of a wastewater plant contained the lowest amount of nutrients (acetate and nitrogen) and had the lowest optical density (OD_750_: 0.02) compared to the 10 v/v % fermentation effluent (FE) originated from a production scale biogas digester (OD_750_: 0.72) and to the 5 v/v % chicken manure supernatant (CMS: OD_750_: 0.25) (Supplementary Information). The nutrient removal rate of phosphate and total nitrogen (mostly ammonium) was also shown to be dependent on the light penetration. The highest phosphate removal rate was observed in CMS (0.20 mM day^–1^), while only 0.02 mM day^–1^ and 0.01 mM day^–1^ phosphate uptake were monitored in MW and in FE, respectively (Figure 1C). The monitored phosphate consumption in CMS were comparable to that of measured in TAP medium (0.20 mM day^–1^). Moreover, in all tested media the microalgal-bacterial consortia removed nitrogen more effectively than phosphate. Total nitrogen removal rate was 0.32 mM day^–1^ in MW, 0.78 mM day^–1^ in FE and 2.46 mM day^–1^ in CMS, respectively (Figure 1E). Similar values were observed for the ammonium content (MW: 0.31 mM day^–1^, FE: 0.77 mM day^–1^) and CMS: 2.44 mM day^–1^) (Figure 1D). Significant organic carbon utilization was observed in all types of liquid wastes. The observed total nitrogen (and ammonium) removal rate were higher in CMS compared to TAP medium (CMS: 2.46 mM day^–1^ and in TAP: 1.31 mM day^–1^, respectively). Carbon removal rate was around 82% in all liquid wastes (CMS: 2.20 mM day^–1^, FE: 1.51 mM day^–1^, MW: 0.38 mM day^–1^) (Figure 1E). Likewise, considerable decrease in total VOAs (and acetic acid) was monitored through the experiment (FE: 2 mM day^–1^, MW and CMS: 3 and 108 mg L^–1^ day^–1^) (Figure 1F). As expected, the high C utilization capability of *C. vulgaris* and its phycosphere is in strong correlation with the BOD consumption (CMS: 78%, FE: 77% and MW: 88%) (Figure 1B). During cultivation pH increase was observed (Figure 1A). The increased pH correlated with the degradation of the organic substrates. The dry mass of the co-cultivated *C. vulgaris* biomass was the highest in CMS with 0.70–0.90 g DM L^–1^ day^–1^, while in FE it was 0.30–0.60 g DM L^–1^ day^–1^. The lowest microalgal-bacterial biomass was measured in MW with a value of 0.10–0.20 g DM L^–1^ day^–1^. The bacterial biomass was only ∼10% of the total biomass in MW, while these values were ∼38 and ∼27% in FE and CMS, respectively (Supplementary Information and Figure 1G). Highest biomass production was observed in CMS followed by TAP, FE and MW (Figure 1G). The cultivated total algal-bacterial biomass carbon to nitrogen ratio in MW, FE and CMS was 9:1, 7:1 and 6:1, respectively. The higher C/N ratio of MW compared to the TAP control (5:1) might indicate nitrogen limitation in MW. The biochemical methane potential (BMP) of the cultivated mixed biomasses show negligible differences compared to the TAP control (TAP: 249 ± 15 CH_4_ mL_*N*_ g oDM^–1^; MW:236 ± 14 CH_4_ mL_*N*_ g oDM^–1^; FE: 238 ± 14 CH_4_ mL_*N*_ g oDM^–1^ and CMS: 241 ± 15 CH_4_ mL_*N*_ g oDM^–1^) (Figure 1H).

### Read-Based Metagenomics Analysis of the Phycosphere

**FIGURE 2:**
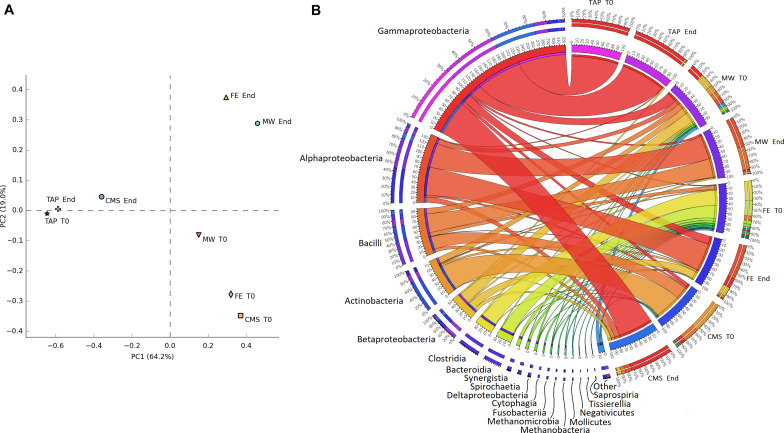
**(A)** PCA of prokaryotic communities in various cultivation media. The variation represented by the first axis (PC1, 64% of overall variation) and the second axis (PC2, 19% of overall variation) indicating diverse phylogenetic structures. **(B)** Relative distribution of abundant microbial classes in different media (left side: classes, right side: cultivation media).

An average of 271,721 sequence reads were generated for each sample, with a mean read length of 231 nucleotides using an Ion Torrent PGM sequencing platform. Sequence reads were quality filtered by Prinseq, this resulted in an average of 266,119 reads with a mean length of 232 nucleotides (Supplementary Table 1). The sequences were analyzed and bacterial partners of *C. vulgaris* were identified using the Kaiju software on Progenomes2 database. The comparison of the prokaryotic microbes using PCA showed significant community shifts between the different wastewater samples over cultivation time (Figure 2A). At the start point (T0) the CMS, FE and MW liquid wastes have diverse microbial community (Figure 2B). The most abundant classes in CMS were *Actinobacteria* (55%), *Bacilli* (27%) and *Gammaproteobacteria* (7%), while in FE *Clostridia* (33%), *Bacteroidia* (27%), *Bacilli* (8%), and in MW *Beta*- and *Gammaproteobacteria* (23–23%) as well as *Actinobacteria* (13%) dominated. The relative abundance of the observed taxa significantly changed over the cultivation period. The *Alpha*-, *Beta*- and *Gammaproteobacteria* and *Bacilli* classes dominated the prokaryotic community at the end point of the experiments (CMS: *Gammaproteobacteria* 74%, *Alphaproteobacteria* 11%, *Betaproteobacteria* 7%; FE: *Alphaproteobacteria* 60%, *Gammaproteobacteria* 17%, *Betaproteobacteria* 16%; MW: *Alphaproteobacteria* 52%, *Bacilli* 40%, *Gammaproteobacteria* 4%, respectively). The control TAP media showed the least microbial shift between the start and the end of the cultivation, where representatives of the *Gammaproteobacteria* class (T0: 100%; end: 95%, respectively), were the most abundant (Figures 2A,B).

### Genome-Centric Analysis of the Phycosphere

Metagenome assembly was carried out by Megahit. A total of 6,148 contigs with a minimum length of 2,000 nucleotides were generated. The contigs were then binned together using MAXBIN2, METABAT2 and CONCOCT automated binning programs. The generated bins were further refined by human guided binning process based on automated binning results with Anvi’o. The 7 bins accounted for a total of 20,038,573 nucleotides. Bins were checked for completion and contamination using CheckM.

**FIGURE 3:**
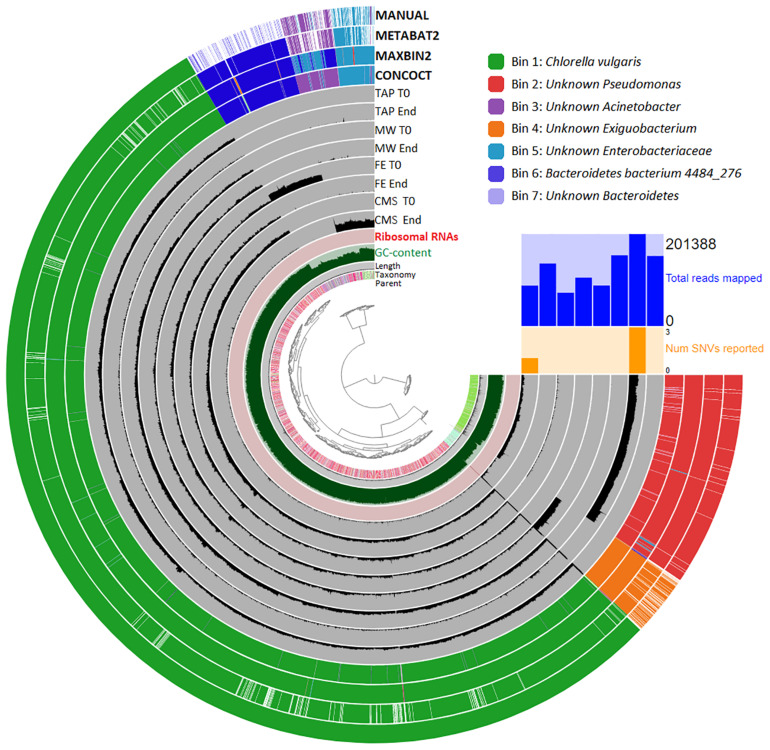
Visualization of the genome-centric metagenomics data. The hierarchical dendrogram of the contigs based on its tetra-nucleotide frequency is in the center of the picture. The taxonomy of the individual contigs is color-coded. The length, GC content, and the presence of ribosomal RNAs of contigs is displayed at the next level. The inner circles show the frequency of the contigs in each wastewater type (CMS: chicken manure supernatant, FE: fermentation effluent, MW: municipal wastewater) in time (T0: start point, End: end point). The outer layer shows the CONCOCT, MAXBIN2, METABAT2 and manual binning results (color-code: upper right corner).

Seven metagenome assembled genomes (MAGs) were generated by Anvi’o (Figure 3). Bin 1 contained the *C. vulgaris* genome fragments. Beside Bin 1 six bacterial MAGs were detected. From these six MAGs five belonged to partly unknown taxa, namely *Pseudomonas*, *Exiguobacterium*, *Acinetobacter*, *Enterobacteriaceae* and *Bacteroidetes*. The *unknown Pseudomonas* (Bin 2) showed a high degree of genome completeness (95%). This MAG included ribosomal maturation proteins (Supplementary Table 2), however, 16S rRNA sequences were not found by HMMER (Bowers et al., 2017). One species level bin (Bin 6) belonged to the *Bacteroidetes bacterium 4484_276*. By mapping back the original reads to the *unknown Pseudomonas* (Bin 2) and *unknown Acinetobacter* (Bin 3) bins it was observed, that these microbes were detected in all cultivation media at each time point. The *unknown Enterobacteriaceae* (Bin 5) was found in all liquid waste cultivations (i.e., MW, FE, CMS), while the *unknown Exiguobacterium* (Bin 4) occurred only in MW. The low quality *Bacteroidetes bacterium 4484_276* (Bin 6) and the *unknown Bacteroidetes* (Bin 7) bins were detected only in FE.

To predict protein pathways, the translated protein coding sequences created by Prokka were further analyzed to calculate module completion ratio (MCR) by MAPLE 2.3.2 using the Kegg database (Kanehisa and Guto, 2000; Seemann, 2014; Arai et al., 2018). The *unknown Pseudomonas* (Bin 2) bin genom harbored complete pathways of gluconeogenesis, Entner-Doudoroff pathway, pyruvate-oxidation, beta-oxidation, sulphate reduction, pentose phosphate pathway, fatty acid, amino acid, cofactor and vitamin metabolism (Supplementary Table 2). The MCR of vitamin B biosynthesis was also found at high percentage in the *unknown Pseudomonas* MAG. Among vitamin B variants, the complete biotin (B_7_) biosynthesis pathway was detected (100%) in Bin 2, while the completeness of cobalamin (B_12_) and thiamin (B_1_) biosynthesis pathways were 86% and 60%, respectively. Between the MAGs showing low degree of genome completeness the *unknown Acinetobacter* (Bin 3) and the *unknown Enterobacteriaceae* bin (Bin 5) had complete MCRs for acetate kinase pathway, while the *unknown Exiguobacterium* (Bin 4) and *Bacteroidetes bacterium 4484_276* (bin 6) bins had complete phospho-ribose-diphosphate pathway. The *unknown Bacteroidetes* (Bin 7) had the lowest genome completeness among the detected MAGs, therefore complete pathways could not be detected in this bin (Supplementary Table 2).

## Discussion

Microalgae and their phycosphere represent powerful natural associations, which can be exploited in bioremediation and biofuel production (Gonçalves et al., 2017; Guldhe et al., 2017). Using liquid wastes for alternative algae cultivation has emerged as a potential cost effective strategy to make microalgae biotechnology more sustainable and economically feasible. It is essential to understand the nature of microalgal-bacterial relationships in order to develop combined bioremediation and biofuel production systems. Therefore, the main objective in this study was the assessment of nutrient removal and microalgal-bacterial biomass production efficiency using different types of wastewater sources (i.e., chicken manure supernatant, fermentation effluent and municipal wastewater). Furthermore, bioremediation and production efficiency data were supported by applying read-based and novel genome-centric approach for the identification of the phycosphere components and their functional profiles.

### Chlorella Vulgaris and Its Phycosphere Is Effective in Bioremediation of Liquid Wastes

THE following major bioremediation process parameters were measured during the experiments: pH, biomass yield, carbon, nitrogen and phosphorous content. The biomass’ carbon/nitrogen ratio and biochemical methane potential were also characterized. The experiments were designed for 4 days, since previous literature data indicated that *C. vulgaris* entered stationary growth phase by the 4th–5th day, no significant biomass production could be observed thereafter (Mujtaba et al., 2015, 2017; Otondo et al., 2018; Qi et al., 2018) (Supplementary Information).

The total carbon (TC), total nitrogen (TN) and phosphate (PO_4_^3–^) concentrations of the applied liquid wastes substantially varied (Figures 1E,C and Supplementary Information). The major nutrients required for microalgal growth are nitrogen and phosphorus incorporated to the cells via active transport. Ammonium is among the most common forms of nitrogen that can easily be utilized by most microalgal species (Gonçalves et al., 2017). Thus, liquid wastes represent a cheap source of nitrogen for microalgal cultivation (Razzak et al., 2013). Previously it was observed, that the optimal ammonium concentration for microalgal cultivation was around 8–10 mM (Uggetti et al., 2014; Chen et al., 2018), higher concentration might inhibit microalgal growth (Källqvist and Svenson, 2003). Another important element required for microalgae growth and metabolism is phosphorus primarily occurring in the form of phosphate (PO_4_^3–^) in wastewater. Phosphorus is an essential ingredient of ATP and nucleic acids in the cells. Phosphate availability has a large impact on microalgal photosynthesis as well (Razzak et al., 2013). Optimal phosphate concentration was found around ∼1 mM (Chiu et al., 2015). The concentration of ammonium and phosphate were relatively low in the applied non-diluted MW (NH_4_^+^-N: 1.6 mM; PO_4_^3–^: 0.1 mM) (Figures 1C,D and Supplementary Information). In the diluted FE (10 v/v%) the amount phosphate was low (PO_4_^3–^: 0.1 mM), while the ammonium content was approximately half of the optimum (NH_4_^+^-N: 4.8 mM). The diluted CMS (5 v/v%) contained high amount of both nutrients (NH_4_^+^-N: 13.7 mM; PO_4_^3–^: 1.2 mM) (Figures 1C,D and Supplementary Information). The ammonium and phosphate removal rates were also high in CMS (NH_4_^+^-N: 2,44 mM day^–1^; PO_4_^3–^: 0.20 mM day^–1^), while lower in FE (NH_4_^+^-N: 0.77 mM day^–1^; PO_4_^3–^: 0.01 mM day^–1^) and MW (NH_4_^+^-N: 0.31 mM day^–1^; PO_4_^3–^: 0.02 mM day^–1^). The experimental data indicated that mostly *C. vulgaris* was responsible for the removal of ammonium and phosphate, and the biomass yield strongly correlated with the removal efficiencies. The results also implied to the dependency of microalgae growth on the available nitrogen sources, which is in good correlation with previous studies (Chiu et al., 2015). The observed low nitrogen content of the biomass generated on MW compared to the TAP control might be explained by the nitrogen limitation (Klassen et al., 2015; Seger et al., 2019).

Microalgae can fix CO_2_ derived from flue gas emission through photosynthesis (Sayre, 2010; Pires et al., 2012). Additionally, microalgae are able to uptake soluble carbonates as a source of CO_2_ (Thomas et al., 2016; Sydney et al., 2019). This uptake depends on the environmental pH. At low pH values the CO_2_ uptake occurs through diffusion (pH 7 ± 1), while in the case of bicarbonate, which is the common form of inorganic carbon under high pH (10 ± 1), the microalgal cells use active transport (Gonçalves et al., 2017). Microalgal photosynthesis raises pH by consumption of CO_2_ and HCO_3_^–^. It was observed that microalgal growth rate is affected by the pH as pH affects the availability of inorganic carbon. When pH is around or over 10, CO_2_ is limiting and bicarbonate is used as a carbon source (Otondo et al., 2018). The pH is slightly increased during the microalgal-bacterial biomass generation in all type of liquid wastes indicating effective photosynthetic activity of microalgae. At the end point of the biomass production in MW the pH was high, this might have been an inhibitory on microalgal biomass growth beside the limited nutrient source (Figure 1).

Although microalgae are mainly autotrophic, *C. vulgaris* is able to grow in a mixotrophic/photoheterotrophic way using organic carbon source (e.g., acetate, glucose) in addition to CO_2_ (Skorupskaite et al., 2015; Zuñiga et al., 2016). Typically both respiratory and photosynthetic processes occur in darkish wastewater (Morales-Sánchez et al., 2015; Skorupskaite et al., 2015; Zuñiga et al., 2016). Microalgae also consume the CO_2_ released from bacterial respiration, in turn the algae provide the O_2_ necessary for the phycospheric bacteria to degrade organic carbon sources (Fuentes et al., 2016; Liu J. et al., 2017). Therefore, organic carbon source of liquid wastes is readily reduced by both microalgal and bacterial metabolic activities. Furthermore, it was observed earlier that microalgae could improve the energy efficiency of BOD removal (Mujtaba and Lee, 2016). These observations were confirmed, significant carbon loss was detected in all type of applied wastewaters (over 80%), which was in clear correlation with the BOD removal rate.

Using microalgae and its phycosphere to utilize nutrients from wastewater for biomass production and the combined use of the generated biomass for biofuel generation is a promising and promoted way to build circular economy (Chiu et al., 2015; Zhu et al., 2016). The advantage of the algal biomass-based biogas production is that the microalgal-bacterial biomass can be directly applied in the biogas reactor, the total biomass is degraded and converted to methane and CO_2_ by a complex microbial community in a well-controlled manner (Guldhe et al., 2017). Microalgal dry biomass productivity was found to be the most effective in CMS (18% higher compared to TAP) followed by FE (CMS: 0.70–0.90 g DM/L/day; FE: 0.30–0.60 g DM/L/day), while the lowest biomass was detected when using MW (0.10–0.20 g DM/L/day) (Supplementary Information and Figure 1G). Similarly, bacterial content was found to be higher in biomass generated in CMS and FE (27 and 38%), while only 10% in MW. The high nutrient content (including acetate, phosphate and ammonium) of CMS explains its effectiveness in biomass production. The biochemical methane potential (BMP) of the biomass generated in the alternative media were comparable to the methane potential of the biomass produced on TAP control (ranging from 236 to 241 CH_4_ mL_*N*_/g oDM in CMS, FE, and MW, while 249 ± 15 CH_4_ mL_*N*_/g oDM in TAP). Differences in BMP might be caused by the biomass carbon to nitrogen ratio and by bacterial content of the biomass (Arcila and Buitrón, 2016; Molinuevo-Salces et al., 2016; Jankowska et al., 2017). The presence of bacteria also explains the relatively higher C/N ratio of biomass cultivated in FE and CMS compared to that of TAP. However, in the aspect of anaerobic digestion this ratios are far from the optimal range (C/N: 20–30:1) (Ward et al., 2014). Thus, the long-term effects of the low C/N ratio and the bacterial content of the biomass on the anaerobic digestion and on the decomposing microbial community need to be further investigated (Wirth et al., 2015a,b, 2018).

### Revealing the Phycosphere of Microalgae Cultivated on Liquid Wastes by Read-Based and Genome-Centric Approach

The read-based metagenomics approach revealed a diverse microbial composition at the start point of cultivations in different type of liquid wastes (Supplementary Information). The PCA of the prokaryotic communities showed significant alterations during the cultivation period (Figure 2A). At the starting point the highest diversity was observed in FE, where *Clostridia*, *Bacteroidia* and *Bacilli* were the most abundant classes. *Beta*,- *Gammaproteobacteria* and *Bacilli* dominated the microbial communities in MW. *Actinobacteria*, *Bacilli* and *Gammaproteobacteria* were the most abundant classes in CMS (Figure 2B). The observed microbial classes are typical for chicken manure, municipal wastewater and anaerobic digesters (Lu et al., 2007; Ju et al., 2014; Campanaro et al., 2020). The starting communities were significantly altered by the end of the cultivation period. Mainly *Alpha*-, *Beta*-, *Gammaproteobacteria* and *Bacilli* became the most dominant classes (Figure 2B). In previous studies similar changes were observed in the prokaryotic microbial community composition in microalgal-seeded systems (Krustok et al., 2015; Chen et al., 2019; Paquette et al., 2020). The TAP medium (control) showed the lowest composition change, in this medium the representatives of *Gammaproteobacteria* class were the dominant bacterial partners of *C. vulgaris* microalgae throughout the cultivation. Two further interesting aspects were observed in the microbial communities. On one hand the prokaryotic community of CMS at the end point was the most similar to that of the TAP medium (Figure 2A). On the other hand the dominance of the class *Gammaproteobacteria* is in close correlation with the biomass yield (Figures 1, 2B).

The genome-centric metagenomics results further explain these interesting observations. The human-guided binning approach resulted one medium (Bin 2) and six low quality (Bin 1, 3–7) Metagenome-Assembled Genomes (MAGs) (Bowers et al., 2017). These bins are identified as one eukaryotic algae MAG (Bin 1) and six bacterial MAGs (Bin 2–7). The *unknown Pseudomonas* (Bin 2), *unknown Acinetobacter* (Bin 3) and *unknown Enterobacteriaceae* (Bin 5) belong to the class *Gammaproteobacteria* within the phylum *Proteobacteria*. Two bins were found as representatives of the phylum *Bacteroidetes*, these are the *Bacteroidetes bacterium 4484-246* MAG (Bin 6) and an *unknown Bacteroidetes* MAG (Bin 7), while the *unknown Exiguobacterium* MAG (Bin 4) belongs to the phylum *Firmicutes* (Figure 3).

Multiple members of the class *Gammaproteobacteria* and the phylum *Bacteroidetes* are considered as Plant Growth Promoting Bacteria (PGPB) interacting with microalgae trough metabolite exchange and by enhancing the microalgal biomass yield and lipid production (Seymour et al., 2017; Calatrava et al., 2018; Cho et al., 2019). The representatives of class *Gammaproteobacteria*, the phylum *Bacteroidetes* and the genus *Exiguobacterium* are commonly found in the phycosphere of *C. vulgaris* cultivated on liquid wastes strengthening the hypothesis, that there are a specific interactions between microalgae and bacteria (Guo and Tong, 2014; Kouzuma and Watanabe, 2015; Mujtaba et al., 2017; Cheah et al., 2018; Qi et al., 2018). It was reported that the representatives of the genus *Pseudomonas* are capable of increasing the growth rate of *Chlorella* microalgae species through the reduction of photosynthetic oxygen tension (Berthold et al., 2019) beside their decomposing activities (Mujtaba et al., 2017; Cheah et al., 2018). The presence of *Pseudomonas* sp. resulted higher *Chlorella* cell concentrations in a given period compared to that observed in axenic microalgae culture (Guo and Tong, 2014; Mujtaba and Lee, 2016). Certain *Pseudomonas* and *Acinetobacter sp.* also promoted the *Chlorella* microalgae growth when cultivated on palm oil mill effluent (Cheah et al., 2018). A symbiotic relationship between *Chlorella* and *Bacteroidetes* species was described recently, the abundance of *Bacteroidetes* specifically increased during pre-treatment of dairy-derived liquid digestate (Zhu et al., 2019). In another study *Proteobacteria* and *Bacteroidetes* induced growth promotion of three microalgae, *Chlamydomonas reinhardtii*, *C. vulgaris* and *Euglena gracilis* in wastewater and swine manure effluent (Toyama et al., 2018). The genus *Exiguobacterium* was previously described among the dominant bacteria during domestic wastewater treatment, this specific bacterium was shown to promote *Chlorella* biomass accumulation and chlorophyll synthesis (Qi et al., 2018; Ren et al., 2019).

The read coverage of bins indicated that the *unknown Pseudomonas* (Bin 2) and *unknown Acinetobacter* (Bin 3) were presented in all types of wastewater media. The *unknown Enterobacteriaceae* (Bin 5) was detected in CMS, FE and MW, while *Bacteroidetes bacterium 4484_276* (Bin 6) and the *unknown Bacteroidetes* (Bin 7) were present only in FE. These data indicated that some of the bacteria were in strong interaction with the *Chlorella* algae while the others were specific to the applied wastewater type. It was reported that many bacteria are able to survive together with microalgae in algae culture collections for long term (Krohn-Molt et al., 2017). The *unknown Pseudomonas* (Bin 2) and the *unknown Acinetobacter* (Bin 3) seem to belong this category, they had a strong interaction with *Chlorella* and might have been inoculated together into the examined waste liquids. The *unknown Enterobacteriaceae* and *Exiguobacterium*, furthermore the representatives of *Bacteroidetes* are likely to be wastewater-specific bacterial strains (Toyama et al., 2018).

Multiple factors influence the presence of bacterial partners of eukaryotic microalgae. A highly important factor is the algal photosynthesis, through which microalgae can increase the dissolved oxygen concentration and the pH of the medium (Seymour et al., 2017). Also the microalgal products having bactericidal effect are important in shaping the phycosphere. The *C. vulgaris* are able to produce a mixture of polyunsaturated fatty acids exhibiting antibiotic activity, i.e., chlorellin (Fergola et al., 2007). Chlorellin is produced in small amount in stationary growth phase, and it exerts different inhibitory effects on different bacteria (DellaGreca et al., 2010; Alwathnani and Perveen, 2017). The effect of chlorellin might have been limited on the development of the phycosphere due to the applied short cultivation time (4 days). Nevertheless, bacteria are also able to influence microalgal growth through nutrient competition (Guldhe et al., 2017). Based on the measurement of the key nutrients and binning results, microalgae and bacteria are competing for VOAs (i.e., acetate). *C. vulgaris* is able to use acetate in photoheterotrophic cultivation mode via active transport (Zuñiga et al., 2016; Huang et al., 2017; Cecchin et al., 2018). The functional profiling of the *unknown Pseudomonas* (Bin 2), *unknown Acinetobacter* (Bin 3) and *unknown Enterobacteriaceae* (Bin 5) resulted in pathways with complete module completion ratio (MCR). These pathways are linked to fatty acid metabolism (Supplementary Table 2). Therefore, it is assumed that these bacteria were mainly responsible for the fatty acid consumption, while the microalgae had only minor role in this metabolic activity. They degrade the fatty acids and release CO_2_ during their metabolic activity, this CO_2_ is consumed by microalgae which in turn produce photosynthetic oxygen essential for the bacteria for fatty acid oxidation. According to MCR calculations the *unknown Exiguobacterium* (Bin 4) and the *Bacteroidetes bacterium 4484-246* (Bin 6) have complete phospho-ribo-biphosphate biosynthesis pathway indicating their carbohydrate metabolic activity. It is not clear, whether these bacteria use the microalgal carbohydrate by-products or possibly degrade algal cell wall components. However, it is very likely that these bacteria also produce CO_2_, thereby increase microalgal photosynthetic activity and growth. Since the genome completeness of these bacteria is low, similarly to the *unknown Acinetobacter* (Bin 3) and the *unknown Enterobacteriaceae* (Bin 5), the knowledge on their detailed roles in the phycosphere is limited.

Vitamins like cobalamin, thiamin, biotin are needed in the lipid biosynthesis pathway in microalgae and higher plants (Croft et al., 2006; Smith et al., 2007). Although *C. vulgaris* is not auxotroph for vitamin B derivatives, the addition of these ingredients still have a positive effect for *Chlorella* growth (Croft et al., 2005). Previous studies involving 306 microalgal species showed that more than half of the examined species (51%) required exogenous cobalamin (vitamin B_12_), 22% required thiamin (vitamin B_1_) and 5% required biotin (vitamin B_7_) for better growth (Croft et al., 2006). It was reported that vitamin supplementation increased the lipid production and intracellular vitamin concentration of the *Chlorella* species, which ultimately resulted in increased growth rate and biomass yield (Fazeli Danesh et al., 2018). It is possible to supply these vitamins by the addition of bacterial partners. It is especially beneficial at industrial scale algae farms to increase sustainability and economic feasibilty. The genome-centric binning results showed that the *unknown Pseudomonas* (Bin 2) showed high MCR for biotin (100%), cobalamin (80%) and thiamin (60%) biosynthesis. The capability of this specific MAG to synthesize these important vitamin B derivatives further supports the close relationship between this bacterium and the *C. vulgaris* microalgae.

## Conclusion

The applied microalgae and its phycosphere effectively reduced the carbon, nitrogen and phosphorus content as well as decreased the BOD of the applied liquid wastes. The nitrogen and phosphorus losses were predominantly caused by the microalgal activity. Nitrogen had the greatest effect on the growth of microalgae, however, the algal consumption of this nutrient depended on the transparency of the medium (light penetration) implying to the significance of the photosynthetic algae growth. The fatty acid content of the liquid wastes was used by both the microalgae and the bacterial partners, however, microalgae had limited importance in this activity. The CO_2_ produced by the phycospheric bacteria was consumed by microalgae and in exchange the photosynthetically produced oxygen was respired by the phycospheric bacteria during the oxidation of organic acids. CMS proved to be the most efficient for microalgal dry mass production, while FE and MW had medium and low efficiency in this term, respectively. However, the lowest bacterial content was detected in the dry biomass grown in MW. Diverse prokaryotic microbial community featured the used liquid wastes at the start point of cultivation, which compositions are typical to the given wastewater type. These were significantly changed at the endpoint. The genom-centric approach revealed that the *unknown Pseudomonas* (Bin 2) and the *unknown Acinetobacter* (Bin 3) strongly interacted with *Chlorella.* Such genome-level investigations may reveal bacterial indicators of culture status, which could be useful for monitoring the health of microalgae in complex bioremediating communities (Seger et al., 2019). The explorations on microalgae-bacteria associations in wastewater contribute to the better understanding of phycosphere activities and help their applications in bioremediation and combined next-generation biofuel production.

## Data Availability Statement

The datasets presented in this study can be found in online repositories. The names of the repository/repositories and accession number (s) can be found in the article/ Supplementary Material.

## Author Contributions

RW designed and performed the bioinformatics analyses and composed the manuscript. BP, TB, GL, and ZB performed the wastewater cultivation experiments and analytical measurements. PS contributed to the metagenome analyses. KK and GM designed the study, composed the manuscript and thoroughly discussed the relevant literature. All authors read and approved the final manuscript.

## Conflict of Interest

The authors declare that the research was conducted in the absence of any commercial or financial relationships that could be construed as a potential conflict of interest.

## Footnotes

1 https://www.gimp.org/

## Supplementary Material

SUPPLEMENTARY INFORMATION Bioremediation data, biomass production and biochemical methane production (BMP) measurements.

TABLE S1 Sequence statistics, read-based and genome-centric data.

TABLE S2 Results of module completion ratio (MCR) calculations.

## Abbreviations

BMP: biochemical methane potential (test)
BOD: biological oxygene demand (test)
C/N: carbon to nitrogen ratio
CMS: chicken manure supernatant (medium)
DM and oDM: dry mass and organic dry mass
FE: anaerobe fermentation effluent (medium)
GHG: Green house gas (emission)
MAGs: metagenome assembled genomes
MCR: module completion ratio
MW: municipal wastewater (medium)
PCA: principal component analysis
PGPB: plant growth promoting bacteria
TAP: tris-acetate-phosphate (medium)
TC and TN: total carbon and total nitrogen
VOAs: volatile organic acids.
