# Supplementary material for: Chlorella vulgaris and Its Phycosphere in Wastewater: Microalgae-Bacteria Interactions During Nutrient Removal
Source: Front Bioeng Biotechnol. 2020 Sep 22;8:557572. doi: 10.3389/fbioe.2020.557572 (PMC7537789; doi:10.3389/fbioe.2020.557572)
Supplement: SUPPLEMENTARY INFORMATION — Bioremediation data, biomass production and biochemical methane production (BMP) measurements. [file Data_Sheet_1.DOCX]

Supplementary Information

For: *Chlorella vulgaris* and its phycosphere in wastewater: microalgae-bacteria interactions during nutrient removal

In the first section cultivation media specific information is summarized and compared to the TAP media used as control during the experiment (measured parameters: dry mass, pH, total phosphate, ammonium, biological oxygen demand, volatile organic acid, acetate, total carbon, total nitrogen, and optical density measurements). Data are given from the start point (in the article is T0) and 4 days later (in the article is mentioned as end) including a middle point (2 days later from start point, which is not included in the article). In case of different types of wastewaters, the figures show all the dilutions are monitored trough the experiment (in the main text only the most efficient ones are discussed: CMS 5 v/v%; FE 10 v/v%; MW 100 v/v%). It can be noted that, dry mass measurements are carried out by centrifugation followed biomass separation and drying, therefore these data have an approximate nature (therefore results are given in an approximate percent in the article).

In the second section biomass per day measurement shows the growth dynamic of the most efficient biomass productions observed in different wastewater dilutions. Calculations were carried out with biomass separation by centrifugation and dry mass measurements.

In the third section the biochemical methane potential is summarized (biomass originated from the most efficient cultivation media after a four-day cultivation period). The produced biomass cumulative methane productions are calculated using the VDI standard (VDI 4630, 2006). Cultivation media preparation and measurement methods are described in the article (Materials and methods).

1. **Media specific information**
   1. *Chicken manure supernatant (CMS) data*

1.1.A. Supplementary Figure: results of pH measurements.

**1.1.B. Supplementary Figure:** total phosphate measurements.

**1.1.C. Supplementary Figure:** ammonium ion measurement data.

**1.1.D. Supplementary Figure:** Results of biological oxygen demand calculations.

**1.1.E. Supplementary Figure:** Volatile organic acid measurements.

**1.1.F. Supplementary Figure:** Acetate concentrations.

**1.1.G. Supplementary Figure:** Results of total carbon measurements.

**1.1.H. Supplementary Figure:** Results of total nitrogen measurements.

**1.1.I. Supplementary Figure:** Optical density measurement data. TAP media was used as blank. Measurement was carried out in 750 nm light wavelength.

**1.1.J. Supplementary Figure:** Produced dry biomass production measurements (calculated in mg/100mL) in all dilutions (v/v%). The first three column represents the second and the second three the fourth day biomass production data, calculated from the start point of cultivation.

- 1. *Fermentation effluent (FE) data*

1.2.A. Supplementary Figure: results of pH measurements.

**1.2.B. Supplementary Figure:** total phosphate measurements.

**1.2.C. Supplementary Figure:** ammonium ion measurement data.

**1.2.D. Supplementary Figure:** Results of biological oxygen demand calculations.

**1.2.E. Supplementary Figure:** Volatile organic acid measurements.

**1.2.F. Supplementary Figure:** Acetate concentrations.

**1.2.G. Supplementary Figure:** Results of total carbon measurements.

**1.2.H. Supplementary Figure:** Results of total nitrogen measurements.

**1.2.I. Supplementary Figure:** Optical density measurement data. TAP media was used as blank. Measurement was carried out in 750 nm light wavelength.

**1.2.J. Supplementary Figure:** Produced dry biomass production measurements (calculated in mg/100mL) in all dilutions (v/v%). The first three column represents the second and the second three the fourth day biomass production data, calculated from the start point of cultivation.

- 1. *Municipal wastewater (MW) data*

1.3.A. Supplementary Figure: results of pH measurements.

**1.3.B. Supplementary Figure:** total phosphate measurements.

**1.3.C. Supplementary Figure:** ammonium ion measurement data.

**1.3.D. Supplementary Figure:** Results of biological oxygen demand calculations.

**1.3.E. Supplementary Figure:** Volatile organic acid measurements.

**1.3.F. Supplementary Figure:** Acetate concentrations.

**1.3.G. Supplementary Figure:** Results of total carbon measurements.

**1.3.H. Supplementary Figure:** Results of total nitrogen measurements.

**1.3.I. Supplementary Figure:** Optical density measurement data. TAP media was used as blank. Measurement was carried out in 750 nm light wavelength.

**1.2.J. Supplementary Figure:** Produced dry biomass production measurements (calculated in mg/100mL) in all dilutions (v/v%). The first three column represents the second and the second three the fourth day biomass production data, calculated from the start point of cultivation.

1. **Growth dynamics of biomass in liquid wastes**

**2. Supplementary Figure:** The most efficient biomass growth dynamics over time (days) in the observed liquid wastes.

1. **Biological methane potential of the produced biomass**

**3. Supplementary Figure:** The cumulative biological methane potential of biomasses cultivated on different wastewaters.
